# Soyasaponins reduce inflammation by downregulating MyD88 expression and suppressing the recruitments of TLR4 and MyD88 into lipid rafts

**DOI:** 10.1186/s12906-020-2864-2

**Published:** 2020-06-03

**Authors:** Junbin Chen, Hidayat Ullah, Zhongdaixi Zheng, Xiangfu Gu, Chuhong Su, Lingyu Xiao, Xinglong Wu, Fei Xiong, Qing Li, Longying Zha

**Affiliations:** 1 Department of Nutrition and Food Hygiene, Guangdong Provincial Key Laboratory of Tropical Disease Research, School of Public Health, Southern Medical University, No.1838 Guangzhou Avenue North, Guangzhou, 510515 People’s Republic of China; 2 Department of Dietetics, Nanfang Hospital, Southern Medical University, No.1838, Guangzhou, 510515 Guangdong People’s Republic of China

**Keywords:** Soyasaponin, Toll-like receptor 4 (TLR4), Myeloid differentiation factor 88 (MyD88), Inflammation, Lipid raft

## Abstract

**Background:**

Previous studies indicate that soyasaponins may reduce inflammation via modulating toll-like receptor 4 (TLR4)/myeloid differentiation factor 88 (MyD88) signaling. However, its underlying mechanisms are still not fully understood.

**Methods:**

Lipopolysaccharide (LPS)-challenged inflamed male ICR mice were intervened by intragastrical administration with 10 and 20 μmol/kg·BW of soyasaponin A_1_, A_2_ or I for 8 weeks. The serum inflammatory markers were determined by commercial kits and the expression of molecules in TLR4/MyD88 signaling pathway in liver by real-time PCR and western blotting. The recruitments of TLR4 and MyD88 into lipid rafts of live tissue lysates were detected by sucrose gradient ultracentrifugation and western blotting. LPS-stimulated RAW264.7 macrophages were treated with 10, 20 and 40 μmol/L of soyasaponin A_1_, A_2_ or I for 2 h. MyD88-overexpressed HEK293T cells were treated with 20 and 40 μmol/L of soyasaponins (A_1_, A_2_ or I) or 20 μmol/L of ST2825 (a MyD88 inhibitor) for 6 h. The expression of molecules in TLR4/MyD88 signaling pathway were determined by western blotting. Data were analyzed by using one way analysis of variance or t-test by SPSS 20.0 statistical software.

**Results:**

Soyasaponins A_1_, A_2_ or I significantly reduced the levels of tumor necrosis factor alpha (TNFα), interleukin (IL)-6 and nitric oxide (NO) in serum (*p* < 0.05), and decreased the mRNA levels of TNFα, IL-6, IL-1β, cyclooxygenase 2 (COX-2) and inducible nitric oxide synthase (iNOS) (*p* < 0.05), the protein levels of myeloid differentiation protein 2 (MD-2), TLR4, MyD88, toll-interleukin1 receptor domain containing adaptor protein (TIRAP), phosphorylated interleukin-1 receptor-associated kinase 4 (p-IRAK-4), phosphorylated interleukin-1 receptor-associated kinase 1 (p-IRAK-1) and TNF receptor associated factor 6 (TRAF6) (*p* < 0.05), and the recruitments of TLR4 and MyD88 into lipid rafts in liver (*p* < 0.05). In LPS-stimulated macrophages, soyasaponins A_2_ or I significantly decreased MyD88 (*p* < 0.05), soyasaponins A_1_, A_2_ or I reduced p-IRAK-4 and p-IRAK-1 (*p* < 0.05), and soyasaponin I decreased TRAF6 (*p* < 0.05). In MyD88-overexpressed HEK293T cells, soyasaponins (A_1_, A_2_ or I) and ST2825 significantly decreased MyD88 and TRAF6 (*p* < 0.05).

**Conclusion:**

Soyasaponins can reduce inflammation by downregulating MyD88 expression and suppressing the recruitments of TLR4 and MyD88 into lipid rafts. This study provides novel understanding about the anti-inflammatory mechanism of soyasaponins.

## Background

Non-communicable chronic diseases (NCDs), such as cardiovascular diseases, diabetes, cancer, etc. are increasingly prevalent and have become the leading causes of mortality worldwide according to the World Health Organization (WHO) reports [1]. Although the pathogenesis of NCDs is diversified, inflammation (especially chronic inflammation) is the common one and plays very important roles in the occurrence and development of NCDs [2]. Reduction of inflammation by pharmaceutical and/or nutritional methods is effective in the prevention and treatment of NCDs [3]. However, controlling inflammation by pharmaceutical methods usually brings adverse effects [4]. In the past several decades, the plant food-borne secondary metabolites (phytochemicals) have attracted intensive research interests because they possess a wide-range of health-promoting bioactivities including anti-inflammation and exhibit great potentials in the prevention of NCDs [5, 6]. Soyasaponins are a group of phytochemicals that are present in soybeans and its products with an average content in soybeans varying from 0.17 to 6.16% [7]. They are oleanane triterpenoid glycosides constructing with a non-polar pentacyclic ring and polar sugar chains, and generally classified into four main groups (A, B, E and DDMP) according to the chemical structure of soyasapogenol [8]. The group A soyasaponins including sixteen members (A_1_, A_2_, A_3_, A_4_, A_5_, A_6_, Aa or acetyl A_4_, Ab or acetyl A_1_, Ac, Ad, Ae or acetyl A_5_, Af or acetyl A_2_, Ag or acetyl A_6_, Ah or acetyl A_3_, A_X_ and A_3_) and group B soyasaponins containing seven main members (B_a_ or V, B_b_ or I, B_c_ or II, B_b_’ or III, B_c_’ or B_X_, IV and B_h_) are naturally the most abundant types in soybean and its related products [8, 9]. Soyasaponins have multiple health-promoting effects such as anticarcinogenic, hypocholesterolemic, hepatoprotective, immunomodulatory, neuroprotective, anticoagulant, and antioxidant bioactivities [7–9].

In recent years, the anti-inflammatory bioactivities of soyasaponins have attracted considerable research interests. Kang et al. (2005) found that total crude soyasaponin extracts containing soyasaponin I and soyasaponin II as major saponins (> 50% of total saponins) inhibited the release of prostaglandin E_2_ (PGE_2_), nitric oxide (NO), tumor necrosis factor alpha (TNFα) and monocyte chemoattractant protein (MCP)-1 in a dose-dependent manner and down-regulated the mRNA/protein expression levels of cyclooxygenase (COX)-2 and inducible nitric oxide synthase (iNOS) by blocking IκB-а degradation in lipopolysaccharide (LPS)-stimulated peritoneal macrophages [10]. Our previous studies showed that three purified soyasaponin monomers (A_1_, A_2_, or I) could inhibit the production of NO and TNFа, the iNOS enzyme activity, and the iNOS mRNA expression in a dose-dependent manner through attenuation of NF-κB activation in LPS-stimulated RAW264.7 macrophages [11]. Lee et al. (2010) also found that soyasaponin I inhibited the production of inflammatory cytokines (TNFа and IL-β), inflammatory mediators (NO and PGE_2_), and inflammatory enzymes (COX-2 and iNOS) by suppressing the phosphorylation of IκB-а and the nuclear translocation of NF-κB in LPS-stimulated macrophages. Moreover, soyasaponin I could significantly reduce inflammatory markers, pro-inflammatory cytokines and NF-κB activation in the colon in 3, 4, 5-trinitrobenzenosulfonic acid (TNBS)-induced colitic mice [12]. Soyasaponin A_b_ not only inhibited NO, PEG_2_, TNFа and IL-1β in LPS-stimulated peritoneal macrophages but also suppressed the expression of COX-2 and iNOS, and activation of NF-κB in TNBS-induced colitic mice. Furthermore, soyasaponin A_b_ weakly inhibited the phosphorylation of ERK, JNK and p38 in LPS-stimulated peritoneal macrophages [13]. Soyasaponin A_3_ and two types of soyasapogenols (B and C) also exhibited anti-inflammatory activities by inhibiting TNFа-induced expression of intercellular adhesion molecule-1 (ICAM-1) in THP-1 human monocytic leukemia cells [14]. Kinjo et al. (2000) reported that soyasaponin III had anti-inflammatory properties by exhibiting in vitro anti-complementary activity [15]. More recently, Lan et al. isolated and identified five new triterpenoid saponins from green vegetable soya beans and found that three of them exhibited moderate anti-inflammatory activities by inhibiting the release of NO in LPS-stimulated RAW264.7 cells [16]. We recently found that soyasaponins (A_1_, A_2_ or I) inhibited inflammation (reduced PGE_2_ production and COX-2 expression) by suppressing the reactive oxygen species (ROS)-mediated activation of the phosphoinositide 3-kinase (PI3K)/protein kinase B (Akt)/ NF-κB signaling pathway [17]. Moreover, soyasaponins (A_1_, A_2_ or I) could reduce inflammation in both liver and white adipose tissue in high fat diet (HFD)-induced obese male C57BL/6 J mice [7]. These studies provide evidences that soyasaponins have anti-inflammatory bioactivities both in vivo and in vitro. Collectively, the molecular mechanisms underlying soyasaponin’s anti-inflammatory bioactivities are associated with the inhibitory modulation on signaling pathway including nuclear factor kappa B (NF-κB) [7, 11, 12, 17], phosphoinositide 3-kinase/protein kinase B (PI3K/Akt) [7, 17] and mitogen activated protein kinases (MAPKs) [13, 18, 19]. The NF-κB and MAPKs signaling pathways are known to be the downstream targets of Toll-like receptor (TLR) signaling pathway including TLR4 [20], which suggests the possible involvement of TLR4 in soyasaponin’s anti-inflammatory mechanism.

TLR4 signaling pathway is a part of innate immunity and one of the most important TLRs in the regulation of inflammation [21]. TLR4 mainly localizes on the cell surface of macrophages and other innate immune cells. It recognizes pathogen-associated molecular patterns (PAMPs) like LPS from Gram-negative bacteria, fusion (F) protein from respiratory syncytial virus, etc. and endogenous ligands including heat-shock proteins, hyaluronic acid, β-defensin 2 and palmitic acid [20, 22]. LPS is one of the best studied immunostimulatory exogenous ligands for TLR4 and can induce both systemic and local tissue inflammation [20]. LPS first combines with the LPS-binding protein (LBP) and then forms a signaling complex with TLR4, myeloid differentiation protein-2 (MD-2), and cluster of differentiation 14 (CD14) to initiate the intracellular signal transduction. The TLR4 intracellular signaling is divided into myeloid differentiation factor 88 (MyD88)-dependent and toll/interleukin 1 receptor domain-containing adaptor inducing IFN-β (TRIF)-dependent (MyD88-independent) pathways [23]. It has been shown that MyD88-dependent pathway is responsible for the expression of pro-inflammatory cytokines, while the MyD88-independent pathway mediates the induction of type 1 interferon-inducible genes [20]. Upon LPS stimulation, MyD88 recruits and activates IL-1 receptor-associated kinase-4 (IRAK-4) which results in the subsequent recruitment, activation and degradation of IRAK-1 [24]. Then, the tumor necrosis factor (TNF) receptor-associated factor 6 (TRAF6) is activated downstream of IRAK4/IRAK1 by forming a complex with ubiquitin-conjugating enzyme 13 (Ubc13) and ubiquitin-conjugating enzyme E_2_ variant 1 isoform A (Uev1A). This further activates transforming growth factor-β-activated kinase 1 (TAK1) [25]. TAK1 then activates both downstream IκB kinase (IKK) and mitogen-activated protein kinase (MAPK) pathway. The activated IKK (а/β/γ) complex phosphorylates the inhibitor of NF-κB (IκB), leading to IκB degradation and NF-κB activation. NF-κB activation thus stimulates the expression of pro-inflammatory cytokines (IL-1β, IL-6, IL-8 and TNFа) [20, 24, 25]. MAPK activation induces transcription factor AP-1 which also stimulates the expression of pro-inflammatory cytokines [26]. The TLR4/MyD88 signaling pathway has been shown to be the target of many phytochemicals with anti-inflammatory bioactivities [21].

Lee et al. (2011) found that soyasaponin Ab inhibited the expression of TLR4 and the phosphorylation of IRAK-1 in both the colon of TNBS-induced colitic mice and the LPS-stimulated peritoneal macrophages. Furthermore, soyasaponin Ab significantly inhibited the binding of LPS to TLR4 on macrophages [13]. Fussbroich et al. (2015) indicated that soyasaponin I not only inhibited TLR4- but also TLR2-induced inflammation, while it had no effect on the expression of TLR4 and TLR2 in LPS-stimulated MUTZ-3 cells [27]. We recently showed that soyasaponin I (Bb) inhibited the recruitment of TLR4, MyD88 and TRIF into lipid rafts in LPS-stimulated RAW264.7 macrophages [18]. Additionally, soyasaponin Bb suppressed the LPS-induced formation of TLR4/MyD88 and TLR4/TRIF complexes in lipid rafts. However it did not affect the total expression levels of TLR4, MyD88 and TRIF [18]. These studies indicate that soyasaponins may modulate TLR4 signaling pathway in the situation of inflammation. However, the modulatory mechanisms of soyasaponins (especially different chemical structures of monomers) on TLR4 signaling pathway are still not fully understood. The objective of this study is to investigate the modulatory effects of three types of soyasaponin monomers (A_1_, A_2_ or I) on TLR4 signaling both in vitro and in vivo.

## Methods

### Reagents and chemicals

Soyasaponin monomers (A_1_, A_2_ or I) were prepared by using the methods as previously described [11]. Antibodies for MD-2 and TRAF6 were purchased from Abcam (Cambridge, MA, USA). Antibody for TLR4 (25) was from Santa Cruz Biotechnology, Inc. (Santa Cruz, CA, USA). LPS and antibodies for TIRAP and flotillin-1 were from Sigma (Saint Louis, MO, USA). Antibody for phospho-IRAK4 (p-IRAK4, Thr345) and phospho-IRAK1 (p-IRAK1, Ser376) were from Bioss (Beijing, China). Antibody for cluster of differentiation 68 (CD68) was purchased from Proteintech (Wuhan, Hubei, China). Antibody for GAPDH was from Good here (Hangzhou, Zhejiang, China). Antibodies for MyD88, β-actin and all secondary antibodies used for western blotting were from Cell Signaling Technology, Inc. (Danvers, MA, USA). TRIZOL reagent was purchased from Invitrogen (Invitrogen, USA).

### LPS-challenged inflammatory mice model

A total of 135 9-weeks old male ICR (Institute of Cancer Research) mice were purchased from Guangdong Medical Lab Animal Center (Certification No. 44007200030072). All animals were housed in standard cages with five mice in each cage in an environment of 21–23 °C and 50–60% humidity on a 12 h/12 h light/dark cycle in the specific pathogen free (SPF) lab animal house. All mice were fed with standard AIN-93G chow diet and allowed ultrapure water ad libitum. The diet was free of soybean and its products in order to avoid containing soyasaponins or soyasapogenols. After 1 week of acclimatization, all mice were randomly allotted to the control group (*n* = 15) and the LPS group (*n* = 120). Mice in the LPS group were intravenously injected with LPS (100 μg/kg·BW) via tail vein once a week for a continuous 8 weeks. Meanwhile, mice in the control group were injected with normal saline (NS) in the same way. At the end of the 8-weeks LPS challenge, blood was collected via tail vein of mice and further prepared as serum. The inflammatory markers (TNFα, IL-1β, IL-6, PGE_2_ and NO) in serum were determined to investigate the inflammatory status of mice. The body weight and feed consumption were recorded weekly.

### Soyasaponins intervention on LPS-challenged inflamed mice

Following LPS challenge for 8 weeks, the intervention trial was then carried out. The mice (*n* = 15) in the control group were still used as a negative control and designated to group 1 (G1), and were intragastrically administered with NS containing 0.5% ethanol (ethanol is the solvent for soyasaponins). All mice (*n* = 120) in the LPS group were randomly divided into eight groups (G2 to G9) with 15 mice in each group (*n* = 15). Mice in G2 were intragastrically administered with NS containing 0.5% ethanol as a positive control. Mice in G3 were intragastrically administrated with 0.1 mg/kg·BW of aspirin. Mice in G4 and G5 were intragastrically administrated with 10 and 20 μmol/kg·BW of soyasaponin A_1_, respectively. Mice in G6 and G7 were intragastrically administrated with 10 and 20 μmol/kg·BW of soyasaponin A_2_, respectively. Mice in G8 and G9 were intragastrically administrated with 10 and 20 μmol/kg·BW of soyasaponin I, respectively. Both aspirin and soyasaponins were dissolved in NS containing 0.5% ethanol. All gavage were administrated once a day (in the afternoon in the animal house) for a total intervention period of 8 weeks. During the intervention trial, the diets and ultrapure water were also provided ad libitum. The body weight and feed consumption were recorded weekly.

After an 8-h overnight fast at the end of intervention trial, mice were weighed and sedated with pentobarbital (50 mg/kg body weight) by peritoneal injection and sacrificed by cervical dislocation. The serum and tissue samples were immediately collected and stored at − 80 °C for further analysis.

### Analysis of inflammatory markers in serum

The inflammatory markers including pro-inflammatory cytokines (TNFα, IL-1β and IL-6) and inflammatory mediators (PGE_2_ and NO) in serum were analyzed. TNFα, IL-1β, IL-6 and PGE_2_ were detected by using ELISA assay with commercial kits (Genetimes ExCell Technology Company, Shanghai, China) following the manufacturer’s instructions. NO was determined by the Griess reaction using a commercial kit purchased from Nanjing Jiancheng Bioengineering Institute (Nanjing, Jiangsu, China).

### Real-time fluorescent quantitative polymerase chain reaction (FQ-PCR)

The mRNA expression of inflammatory markers (TNFα, IL-1β, IL-6, iNOS and COX-2) in liver tissues was detected by using FQ-PCR. Preparation of total RNA, cDNA synthesis, and PCR were operated as described previously [7]. The primers used were as follows: TNFα (forward: 5′-CCACCACGCTCTTCTGTCTA-3′, reverse: 5′-TGGTTTGTGAGTGTGAGGGT-3′), IL-6 (forward: 5′-TTCTTGGGACTGATGCTGGT-3′, reverse: 5′-CAGGTCTGTTGGGAGTGGTA -3′), IL-1β (forward: 5′-TGACGGACCCCAAAAGATGA-3′, reverse: 5′-CTGCTGCGAGATTTGAAGCT-3′), iNOS (forward: 5′-ACCCAAGGTCTACGTTCAGG-3′, reverse: 5′-CGCACATCTCCGCAAATGTA-3′), COX-2 (forward: 5′-CAGGTCATTGGTGGAGAGGT-3′, reverse: 5′-TCAGGGATGTGAGGAGGGTA-3′), β-actin (forward: 5′-GTGGGAATGGGTCAGAAGGA-3′, reverse: 5′-CTTCTCCATGTCGTCCCAGT-3′). Samples were normalized by dividing the quantity of the indicated molecules’ genes by the value of a house-keeping gene (β-actin) in the same sample. Results were presented as mRNA relative expression (folds to the control, which denoted as 1).

### Lipid rafts isolation by sucrose gradient ultracentrifugation

Liver tissues were suspended in 2.1 mL ice-cold MBS buffer [25 mM 2-(N-morpholino)-ethanesulfonic acid (MES, pH 6.5) and 0.15 M NaCl] containing 1% Triton X-100, 1 mM phenylmethylsulfonyl fluoride (PMSF) and 1% protease inhibitor cocktail (Sigma), lysed by homogenization, and then incubated on ice for 30 min followed by centrifugation at 700 rpm, 4 °C for 10 min. The supernatants (2.1 mL) were mixed with 2.1 mL 80% sucrose solution in MBS buffer and then transferred to the bottom of centrifuge tubes. These samples were overlaid with 4.2 mL of 30% sucrose in MBS buffer, followed by 2.1 mL of 5% sucrose in MBS buffer. After the samples were centrifuged at 39,000 rpm for 20 h at 4 °C in a Hitachi P40ST rotor, twelve 0.875 mL fractions were collected from the top of the gradient and transferred into separated tubes. Fractions were precipitated by using trichloroacetic acid method.

### Cell culture

RAW264.7 murine macrophages (ATCC® TIB-71™) and HEK293T cells (ATCC® CRL-11268™) were maintained in DMEM (Gibco, Grand island, NY, USA) containing 10% (v/v) fetal bovine serum (Clark, Richmond, VA, USA) and 1% (v/v) penicillin-streptomycin in a humidified atmosphere containing 5% CO_2_ at 37 °C.

In the LPS challenge experiments, RAW264.7 macrophages were stimulated with 1 μg/mL of LPS for different time (0 min, 10 min, 30 min, 1 h, 3 h, 6 h, 12 h and 24 h). In the soyasaponins intervention experiments, RAW264.7 macrophages were pre-incubated with graded concentrations (10, 20 and 40 μmol/L) of soyasaponin (A_1_, A_2_ or I) for 2 h and then stimulated with 1 μg/mL of LPS for suitable times as determined by previous results.

### Cell transfection

HEK293T cells were transfected with MyD88 flag expression plasmid (Addgene plasmid #13093) or the empty plasmid vector using the Lipofectamine™ 3000 reagent (Invitrogen) by following the recommended protocols. HEK293T cells were transfected for 24 h and then treated with graded concentrations (20 or 40 μmol/L) of soyasaponin (A_1_, A_2_ or I), or 20 μmol/L of ST2825 (a MyD88 dimerization inhibitor) for 6 h.

### Western blotting

Western blotting was performed as previously described [7]. Briefly, mice liver tissues or cells samples were homogenized in RIPA lysis buffer (KeyGEN Biotech) containing 1 mM PMSF, 1% protease inhibitors and 0.8% phosphatase inhibitors. Cell lysates were centrifuged (12, 000 rpm) for 10 min. The lysates (20–40 μg/lane) were subjected to 10% SDS-PAGE gels and electrotransferred to PVDF membranes (Millipore, Billerica, MA, USA). The presence of proteins was detected by immunoblotting with primary antibodies overnight at 4 °C and followed by HRP-conjugated secondary IgG antibody. Immunoreactive bands were developed by using enhanced chemiluminescence and visualized by using the Tanon-5200 imaging system (Shanghai, China).

### Statistical analysis

Statistical analyses were performed using one way analysis of variance (one-way ANVOA) and LSD or Dunnett’s T3 multiple comparison tests or t-test by SPSS (edition 20.0) statistical software (SPSS Inc., Chicago, IL, USA). Results are expressed as Means ± SD. Significant values (*p* < 0.05) were marked with an asterisk (*) or an octothorpe (#).

## Results

### Soyasaponins decrease LPS-induced inflammation in ICR mice

LPS has been shown to induce both systemic and local tissue inflammation in mice [28]. In this study, we challenged the ICR mice with intravenous injection of LPS (100 μg/kg·BW) via tail vein once a week for a continuous 8 weeks to establish a model for mimicking the chronic inflammatory status, and then intervened with soyasaponins. As shown in Table S1, chronic challenge of LPS on ICR mice for 8 weeks significantly increased the inflammatory markers (TNFα, IL-6, PGE_2_ and NO) in serum indicating the production of an systemically inflammatory status (*p* < 0.05). Unexpectedly, the IL-1β could not be detected by commercial ELISA kit in this study. Meanwhile, chronic challenge of LPS did not affect the growth (Fig. S1, A) and food consumption (Fig. S1, B) of mice (*p* > 0.05).

**Fig. 1:**
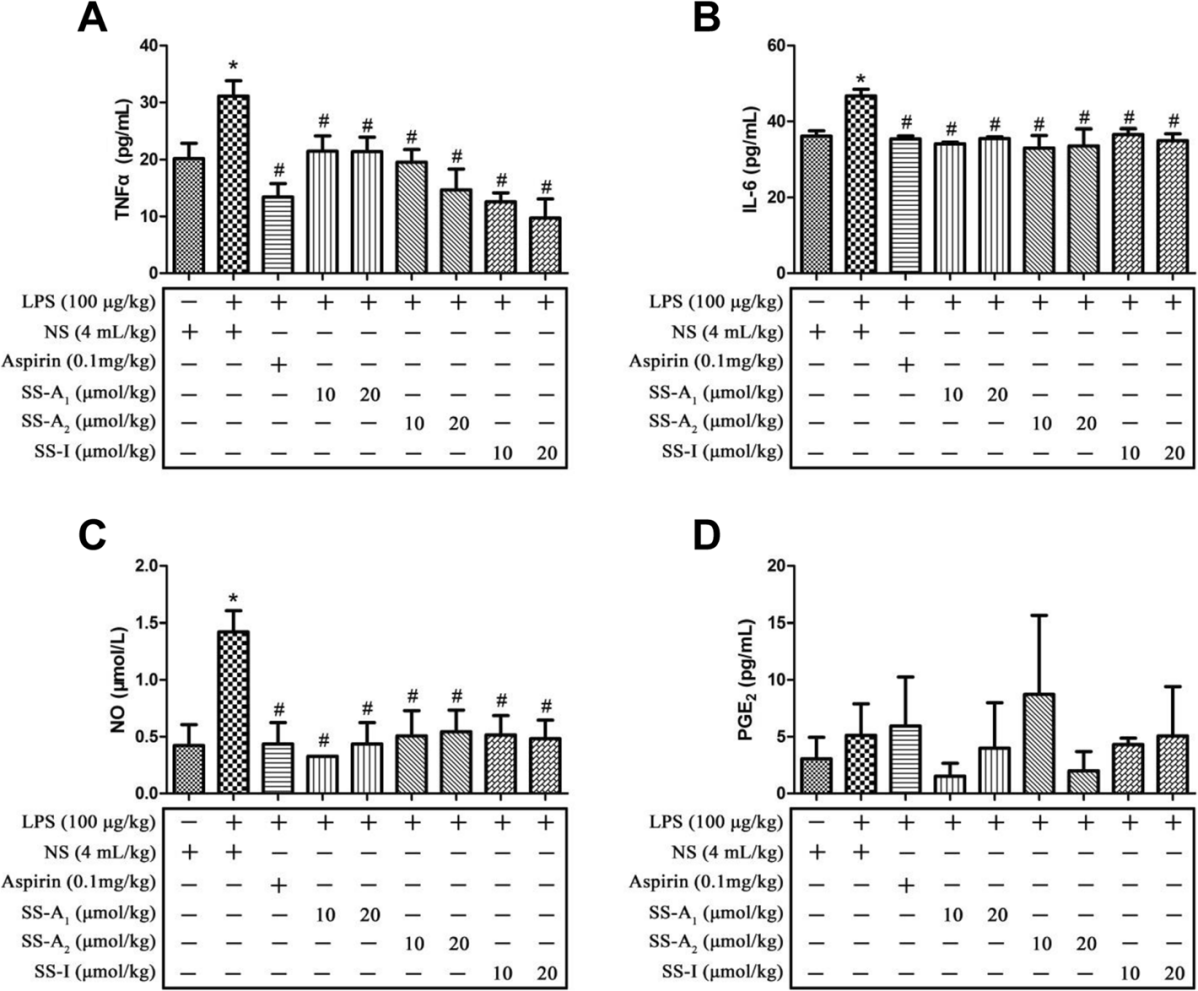
Effects of soyasaponins on inflammatory markers in serum of LPS-challenged ICR mice. The serum levels of TNFα, IL-6, PGE_2_ and NO were determined by commercial ELISA kits in LPS-induced inflammatory mice after intervention by aspirin or soyasaponins (A_1_, A_2_ or I). Results reported are Means ± SD of samples from 9 mice in each group (*n* = 9). Data were statistically analyzed by using one-way ANOVA of SPSS software. *: *p* < 0.05 *v.s.* control, #: *p* < 0.05 *v.s.* LPS alone

Following LPS challenge for 8 weeks, inflamed mice were then intervened by soyasaponins or aspirin for 8 weeks. As shown in Fig. 1, mice in the LPS group had significantly higher levels of TNFα, IL-6 and NO in serum (*p* < 0.05). Both soyasaponins (A_1_, A_2_ or I) and aspirin reduced the inflammatory markers (TNFα, IL-6 and NO) in serum (*p* < 0.05). However, the level of PGE_2_ in serum was not affected (*p* > 0.05). Furthermore, intervention with soyasaponins (A_1_, A_2_ or I) or aspirin for 8 weeks did not change the growth (Fig. S1 C) and feed consumption (Fig. S1 D) of mice (*p* > 0.05).

We further investigated the mRNA expression of inflammatory markers in liver tissues of mice. As shown in Table S2, mice in the LPS group had significantly higher mRNA expression of inflammatory markers (TNFα, IL-6, IL-1β, COX-2 and iNOS) in liver of ICR mice (*p* < 0.05). Intervention with soyasaponins (A_1_, A_2_ or I) or aspirin for 8 weeks significantly reduced the LPS-increased mRNA expression of all these inflammatory markers (*p* < 0.05).

Together, these results indicate that long-term challenge with low-dosage of LPS can induce both systemic (in blood) and local (in liver tissues) inflammation in ICR mice and soyasaponins intervention can decrease the LPS-induced inflammation.

### Soyasaponins inhibit TLR4/MyD88 signaling in liver of LPS-challenged mice

**Fig. 2:**
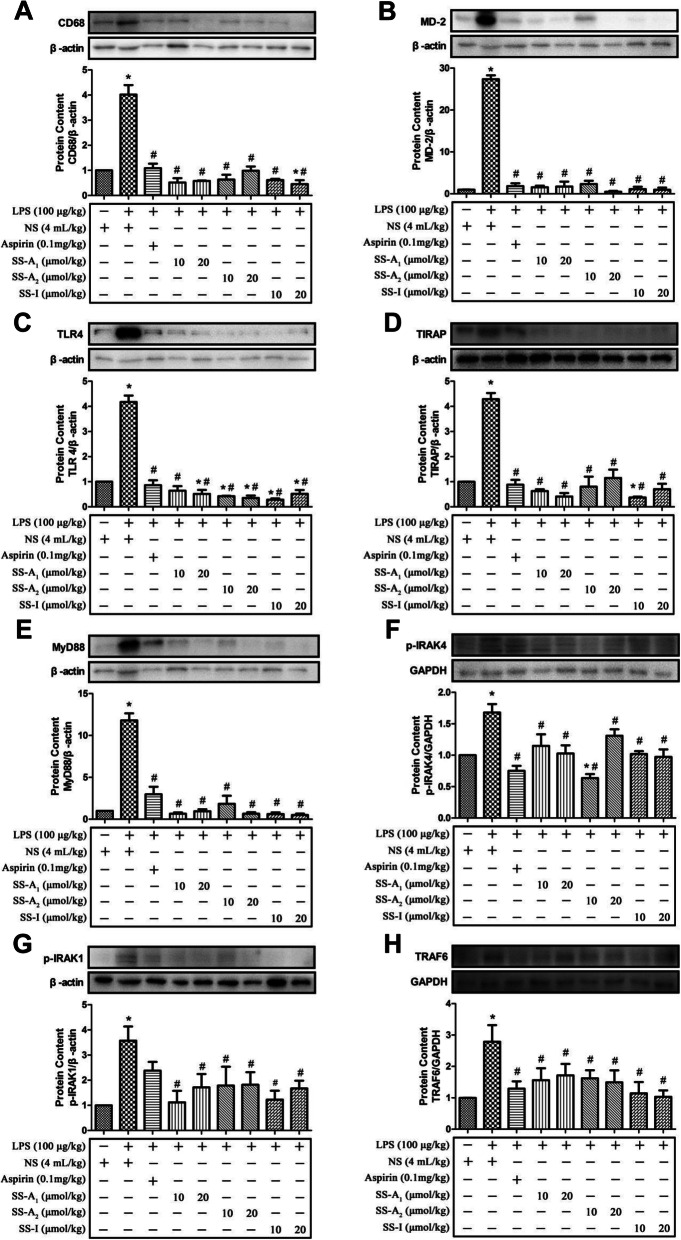
Soyasaponins reduced protein levels of molecules in TLR4/MyD88 signaling pathway and CD68 in the liver tissues of LPS-challenged mice. The protein levels of MD-2, TLR4, MyD88, TIRAP, p-IRAK4, p-IRAK1 and TRAF6 in TLR4/MyD88 signaling pathway, and CD68, a macrophage marker, were measured by western blotting. Results reported are Means ± SD of samples from 6 mice in each group (*n* = 6). Data were statistically analyzed by using one-way ANOVA of SPSS software. *: *p* < 0.05 *v.s.* control, #: *p* < 0.05 *v.s.* LPS alone

TLR4/MyD88 signaling plays primary role in LPS-induced inflammation and has been shown to be the target of many phytochemicals exhibiting anti-inflammatory bioactivities [28]. Here, we analyzed the TLR4/MyD88 signaling pathway in liver tissues of mice. As shown in Fig. 2, LPS challenge on mice activated TLR4/MyD88 signaling in liver as evidenced by increased expression of MD-2, TLR4, MyD88, TIRAP and TRAF6, and enhanced phosphorylation of IRAK-4 and IRAK-1 (*p* < 0.05). Intervention of soyasaponins (A_1_, A_2_ or I) on LPS-challenged mice significantly decreased the expression of MD-2, TLR4, MyD88, TIRAP and TRAF6, and reduced the phosphorylation of IRAK-4 and IRAK-1 (*p* < 0.05) in liver tissues suggesting the inhibitory effects of soyasaponins on TLR4/MyD88 signaling. Similarly, aspirin intervention on LPS-challenged mice also significantly decreased the expression of MD-2, TLR4, MyD88, TIRAP and TRAF6, and phosphorylation of IRAK-4 (*p* < 0.05) in liver. However, aspirin did not significantly affect the phosphorylation of IRAK-1 (*p* > 0.05). CD68 is specifically expressed in macrophages and used as a marker of macrophages’ response to inflammation [22, 29]. Here, LPS challenge significantly increased the CD68 expression in liver tissues indicating the macrophage-associated activation of inflammation there. Both soyasaponins (A_1_, A_2_ or I) and aspirin significantly decreased the expression of CD68 in liver as compared with the LPS alone group (*p* < 0.05). These results show that soysasaponins can inhibit the LPS-induced activation of TLR4/MyD88 signaling in mice liver.

### Soyasaponins inhibit LPS-induced recruitments of TLR4 and MyD88 into lipid rafts of liver tissue lysates

**Fig. 3:**
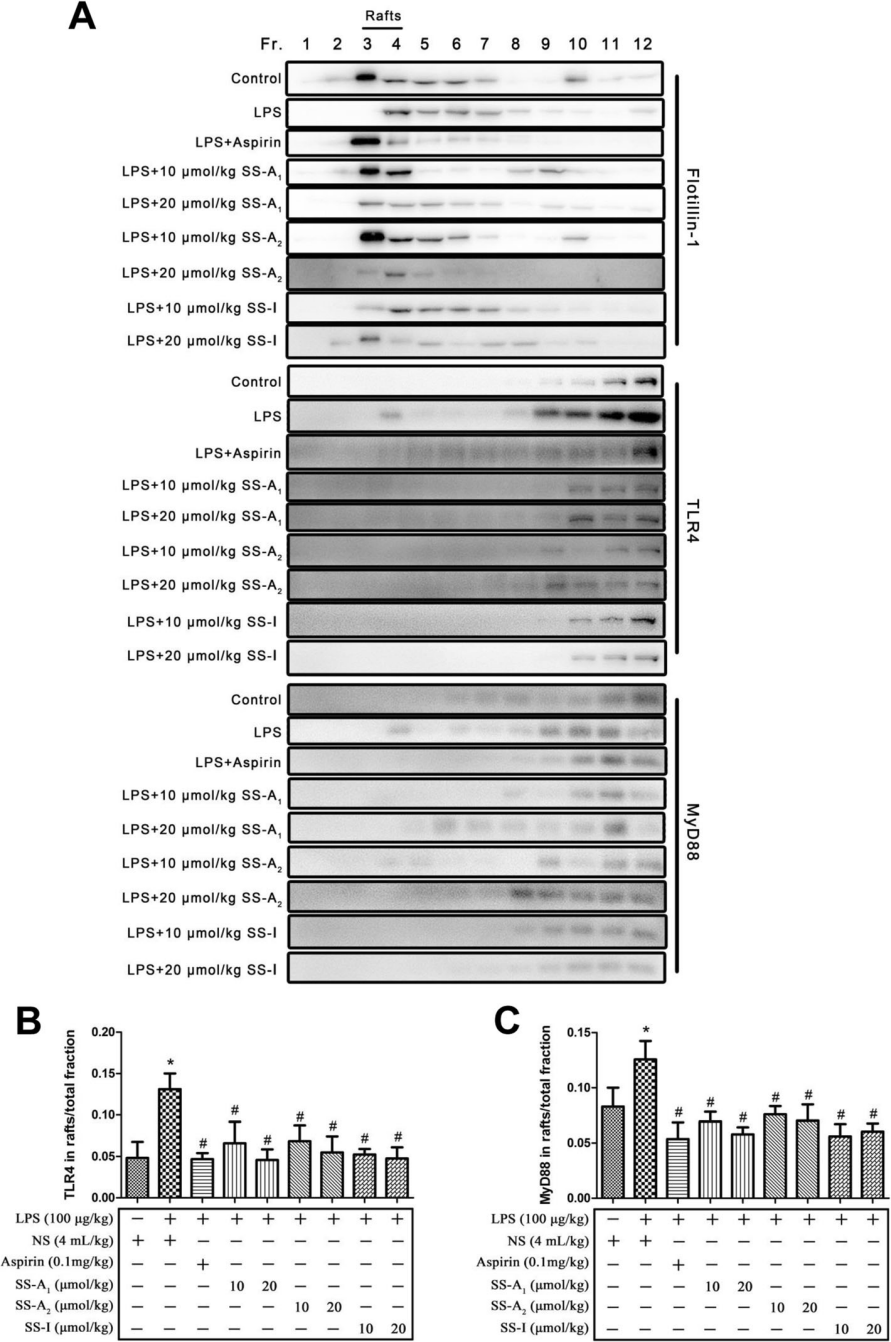
Soyasaponins inhibited the recruitments of TLR4 and MyD88 into lipid rafts in liver tissues. The lipid rafts (fraction 3 and 4) were fractionated from liver tissue lysates by using sucrose gradient ultracentrifugation. The protein levels of flotillin-1 (a lipid raft marker), TLR4 and MyD88 were detected by western blotting. The ratio of the amount of TLR4 (or MyD88) in lipid raft to that in total fractions were quantitatively calculated. Results reported are Means ± SD of samples from six mice in each group (*n* = 6). Data were statistically analyzed by using one-way ANOVA of SPSS software. *: *p* < 0.05 *v.s.* control, #: *p* < 0.05 *v.s.* LPS alone

Lipid rafts have been shown to be essential for the activation of TLR4/MyD88 signaling [30]. We previously found that soyasaponins could reduce inflammation by inhibiting the recruitments of TLR4 and MyD88 into lipid rafts in murine macrophages in vitro [18]. To further address the potential anti-inflammatory mechanism of soyasaponins in vivo, here we analyzed the recruitments of TLR4 and MyD88 into lipid rafts in liver tissues of LPS-challenged mice. As shown in Fig. 3a, flotillin-1, the marker of lipid rafts, was highly rich in fractions 3 and 4 of ultracentrifugation samples of liver tissues. As compared to the control, LPS challenge increased the recruitments of TLR4 and MyD88 into lipid rafts (mainly in fractions 3 and 4) of liver tissues (Fig. 3 a-c) (*p* < 0.05). Treatment of both soyasaponins (A_1_, A_2_ or I) and aspirin significantly reduced the LPS-increased levels of TLR4 and MyD88 in fractions 3 and 4 of liver tissues (*p* < 0.05) indicating the in vivo inhibitory bioactivities of soyasaponins on recruitments of these two molecules into lipid rafts (Fig. 3 a-c).

### Soyasaponins inhibit TLR4/MyD88 signaling in LPS-stimulated murine macrophages

It is known that LPS-stimulated macrophages serve as a good in vitro cell model to investigate the TLR4/MyD88 signaling-mediated inflammation [20, 31]. Here we used LPS-stimulated murine RAW264.7 macrophages to further understand the modulation of soyasaponins on TLR4/MyD88 signaling in vitro.

Firstly, we stimulated RAW264.7 macrophages with 1 μg/mL of LPS for different time (0 min, 10 min, 30 min, 1 h, 3 h, 6 h, 12 h, and 24 h) to understand the time-dependent change rule of protein levels of molecules in TLR4/MyD88 signaling pathway. As seen in Fig. S2, LPS stimulation for 10 min to 24 h did not produce significant change of the protein levels of MD-2, TLR4 and TIRAP in macrophages (*p* > 0.05) (Fig. S2 E). However, LPS treatment for 1 h or 3 h significantly increased the expression levels of MyD88 (*p* < 0.05) (Fig. S2 A). LPS stimulation for 3 h significantly increased the phosphorylation of IRAK4 (*p* < 0.05) (Fig. S2 B). LPS challenge for 3 h, 6 h or 12 h significantly increased the phosphorylation of IRAK1 (*p* < 0.05) (Fig. S2 C). Interestingly, LPS treatment for 30 min to 6 h significantly increased the levels of TRAF6 (*p* < 0.05), but LPS stimulation for 12 h or 24 h decreased the levels of TRAF6 (*p* < 0.05) (Fig. S2 D). These results indicated that LPS stimulation for as short as 30 min could activate TLR4/MyD88 signaling in murine macrophages. Therefore, 30 min was chosen as the starting time duration for LPS challenge in the next step experiment of soyasaponins intervention.

**Fig. 4:**
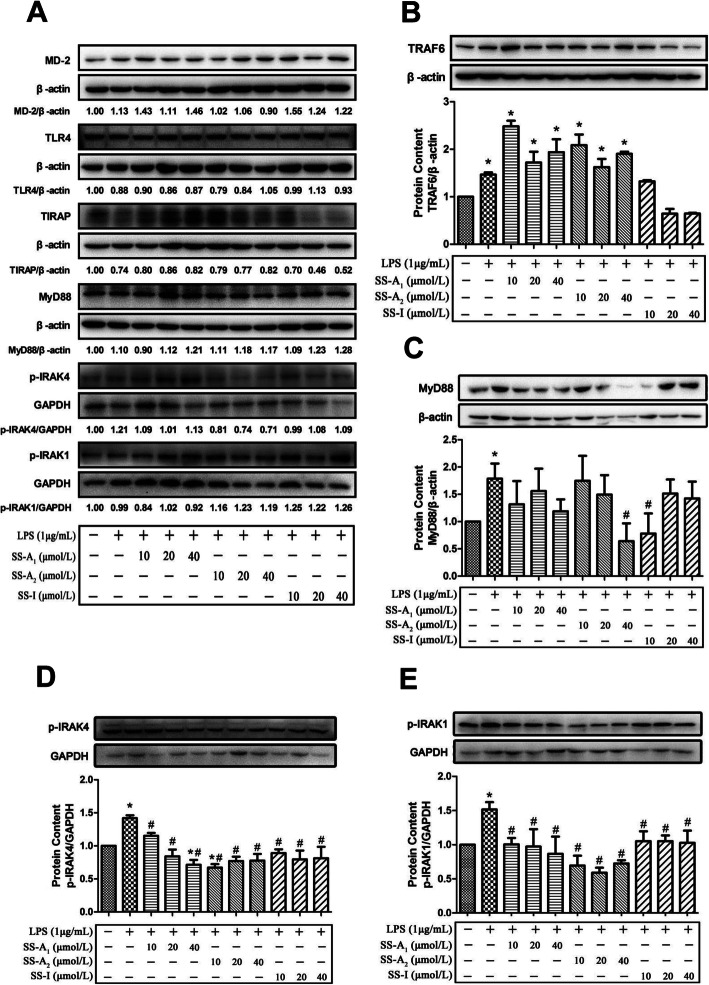
Effects of soyasaponins on the protein levels of molecules in TLR4/MyD88 signaling pathway in LPS-stimulated RAW264.7 macrophages. RAW264.7 macrophages were pre-treated with graded concentrations (10, 20 or 40 μmol/L) of soyasaponins (A_1_, A_2_, or I) for 2 h and then stimulated with LPS (1 μg/mL) for 30 min (**a** and **b**), 1 h (**c**), or 3 h (**d** and **e**). The levels of molecules (MD-2, TLR4, TIRAP, MyD88, p-IRAK4, p-IRAK1 and TRAF6) in TLR4/MyD88 signaling pathway were measured by western blotting. Results reported are Means ± SD of three independent experiments. Data were statistically analyzed by using one-way ANOVA of SPSS software. *: *p* < 0.05 *v.s.* control, #: *p* < 0.05 *v.s.* LPS alone

Secondly, we pre-incubated the murine macrophages with graded concentrations (10, 20 or 40 μmol/L) of soyasaponins (A_1_, A_2_, or I) for 2 h, and then added 1 μg/mL of LPS to stimulate the cells for 30 min, 1 h or 3 h, and detected the protein expression levels of molecules in TLR4/MyD88 signaling pathway by western blotting. As shown in Fig. 4a, LPS stimulation on macrophages for 30 min did not change the protein levels of MD-2, TLR4, TIRAP, MyD88, p-IRAK4 and p-IRAK1. This is in agreement with the above results of time-dependent experiments. Soyasaponins pre-incubation produced no effect on the levels of MD-2, TLR4, TIRAP, MyD88, p-IRAK4 and p-IRAK1 in LPS (for 30 min)-challenged macrophages. LPS stimulation for 30 min significantly increased the level of TRAF6, while this LPS-induced increase of TRAF6 was blunted by pre-treatment of soyasaponin I, but not by soyasaponins A_1_ and A_2_ (Fig. 4b). LPS stimulation for 1 h significantly increased the level of MyD88, which was blocked by pre-incubation of soyasaponin A_2_ (40 μmol/L) and soyasaponin I (10 μmol/L) (Fig. 4b). LPS stimulation for 3 h significantly increased the phosphorylation of IRAK4 (Fig. 4c) and IRAK1 (Fig. 4d) which was blocked by pre-incubation of all soyasaponins (A_1_, A_2_ or I). These results show that soyasaponins can inhibit the TLR4/MyD88 signaling by downregulating the molecule expressions in LPS-stimulated murine macrophages.

### Soyasaponins inhibit the expression of MyD88 and TRAF6 in MyD88-transfected HEK293T cells

**Fig. 5:**
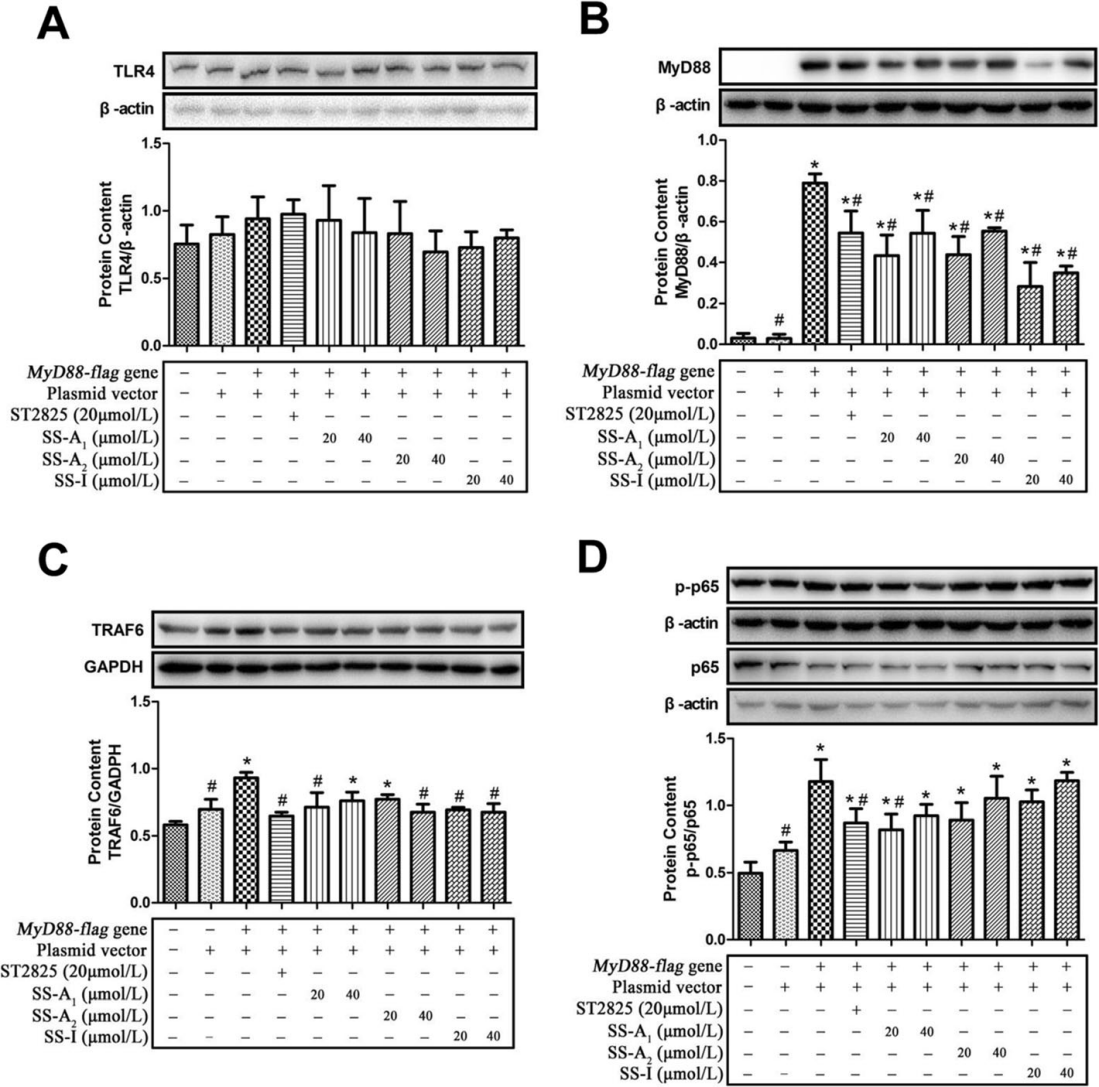
Soyasaponins inhibit the protein expression of MyD88 and TRAF6, and activation of NF-κB in MyD88-transfected HEK293T cells. HEK293T cells were transfected with MyD88-flag plasmid for 24 h, and then treated with graded concentration (20 or 40 μmol/L) of soyasaponin (A_1_, A_2_ or I) or ST2825 (a MyD88 inhibitor) for 6 h. The protein levels of TLR4 (**a**), MyD88 (**b**), TRAF6 (**c**), p-p65 and p65 (**d**) were measured by western blotting. Results reported are Means ± SD of three independent experiments. Data were statistically analyzed by using one-way ANOVA of SPSS software. *: *p* < 0.05 *v.s.* control, #: *p* < 0.05 *v.s.* MyD88-flag plasmid transfected group

Based on the above results in LPS-stimulated murine macrophages, MyD88 was the first upstream molecule that was modulated by soyasaponins in TLR4/MyD88 signaling pathway suggesting MyD88 might be the key target of soyasaponins. Therefore, we applied MyD88 overexpression cell model to further understand the modulation of soyasaponins on TLR4/MyD88 signaling. Human embryonic kidney (HEK) 293 T cells normally express low levels of TLR4 and MyD88 [32]. As shown in Fig. 5, transfection of MyD88 plasmid in HEK293T resulted in high expression levels of MyD88, and also increased the expression of TRAF6, and activated the downstream NF-κB as evidenced by increased ratio of phosphorylated p65 (p-p65) to p65. However, MyD88 plasmid transfection did not affect the expression of upstream molecule of TLR4 in HEK293T cells (*p* > 0.05). Soyasaponins (A_1_, A_2_, or I), similar to ST2825 (the MyD88 inhibitor), significantly decreased the MyD88 plasmid transfection-induced increase of MyD88 expression levels in HEK293T cells (*p* < 0.05). Furthermore, soyasaponin A_1_ (20 μmol/L), A_2_ (40 μmol/L), I (both 20 and 40 μmol/L) and ST2825 significantly decreased the TRAF6 levels (*p* < 0.05). Meanwhile, soyasaponin A_1_ (20 μmol/L) and ST2825 significantly reduced the activation of NF-κB (decreased the ratio of p-p65 to p65) (*p* < 0.05). Neither soyasaponins (A_1_, A_2_, or I) nor ST2825 affected the TLR4 level in MyD88 plasmid-transfected HEK293T cells (*p* > 0.05). Together, these results show that soyasaponins (A_1_, A_2_, or I) can inhibit the expression of MyD88 and TRAF6 in MyD88-transfected HEK293T cells.

## Discussion

Previous studies indicate that soyasaponins may reduce inflammation by regulating TLR4/MyD88 signaling pathway. However, the underlying mechanisms are not fully understood. This study made some novel understanding about the regulation of soyasaponins on TLR4/MyD88-mediated inflammation.

First, results from this study further support the in vivo anti-inflammatory bioactivities of soyasaponins. In recent years, many studies have demonstrated that soyasaponins exhibit anti-inflammatory bioactivities by reducing the pro-inflammatory cytokines and/or mediators (TNFа, IL-1β, IL-6, MCP-1, iNOS, NO, COX-2 and PGE_2_) [7–14, 16–19]. However, only a few of investigations provided in vivo evidences. Lee et al. (2010) demonstrated that oral administration of soyasaponin I (10 and 20 mg/kg) to TNBS-induced colitic mice significantly reduced inflammatory markers, colon length, myeloperoxidase, lipid peroxide (malondialdehyde and 4-hydroxy-2-nonenal), pro-inflammatory cytokines and NF-κB activation in the colon [12]. In 2011, the same lab reported that oral administration of soyasaponin Ab (10 and 20 mg/kg) also inhibited colon shortening, myeloperoxidase activity, the expression of COX-2 and iNOS, and activation of NF-κB in TNBS-induced colitic mice [13]. These two studies showed that soyasaponins (I and Ab) can ameliorate colitis indicating their bioactivities to inhibit inflammation in vivo. It is known that the exertion of physiological functions of phytochemicals in vivo is largely dependent on its absorption and bioavailability. Soyasaponins can be metabolized and degraded to its aglycones (soyasapogenols) by gut microbes [29, 33]. However, both soyasaponin I and its aglycone (soyasapogenl B) have limited absorption by Caco-2 intestinal cells and limited bioavailability in women [29]. In rats, chickens and mice after oral administration, neither soyasaponins nor soyasapogenols were found in the urine or blood based on thin-layer chromatography (TLC) and hemolysis assays [33]. Yoshikoshi et al. (1995) also indicated that neither soyasaponins nor its aglycones (soyasapogenols) were detected in the blood or urine of rats fed with a soybean hypocotyl diet [34]. However, more recently, group B soyasaponins (I and V), deacetyl-soyasaponin A_1_ and soyasapogenols (A and B) could be detected in human or rat serum by using highly sensitive analytical method of high-performance liquid chromatography coupled with electrospray ionization tandem mass spectrometry (HPLC-MS/MS) [35, 36]. These studies suggest that oral dosing of soyasaponins and soyasapogenols can be absorbed but its absorption efficiency is low. Therefore, soyasaponins may probably exert anti-inflammatory bioactivities in vivo through direct or indirect mechanism or both. The direct mechanism is that soyasaponins may be absorbed into the blood and transported to the targeted organs or tissues to reduce inflammation therein. The indirect mechanism is that soyasaponins may antagonist inflammation through exerting bioactive effects in the intestine (especially in the colon) without absorption. It seems that the in vivo anti-inflammatory bioactivities of soyasaponins are to a large extent via the indirect mechanism because the absorption and bioavailability of soysaponins is limited [29]. The two studies from Lee et al. found that soyasaponins exerted anti-inflammatory effects in the colon, which suggests that soyasaponins (or most of them) may go directly into the colon, be metabolized to its aglycones (if not all, at least some of them) and play bioactive roles therein [12, 13]. We recently showed that oral administration of soysaponins (A_1_, A_2_ or I) reduced both systemic inflammation and local tissue (hepatic and adipose) inflammation in HFD-induced obese mice [7]. In that study, evidences supported the indirect mechanism of in vivo anti-inflammatory abilities of soyasaponins, i.e. decreased the lipids accumulation in liver and adipose tissues by promoting its fecal excretion and decreasing intestinal absorption. However, that study did not exclude the possibility that soyasaponins were directly absorbed and transported to the liver and adipose tissues to exert anti-inflammatory activities. In the present study, we used an inflammatory model of ICR mice with intravenous injection of LPS via tail vein to investigate the in vivo anti-inflammatory bioactivities of soyasaponins. We found that oral administration of soysaponins (A_1_, A_2_ or I) could reduce both systemic inflammation (decreased levels of TNFα, IL-6 and NO in serum) and local inflammation in liver (decreased mRNA expression of TNFα, IL-6, IL-1β, COX-2 and iNOS in liver tissues). Inflammation induced by intravenous injection of LPS via tail vein means that the gastrointestinal environment is not involved during the development of systemic and liver inflammation. Thus, this study indicates that soyasaponins may be absorbed into the blood, transported to the liver, and exert anti-inflammatory functions therein. Of course, the absolutely direct evidence is to define the absorptive and distributive concentrations of soyasaponins in blood and organs (e.g. liver). However, the quantification of trace amount of soyasaponins in blood and tissues is still challengeable. Such study is currently on the way in our lab. A study from Hong et al. showed that oral administration of soyasaponins Ab and Bb prevented scopolamine-induced memory impairment in mice by increasing brain-derived neurotrophic factor (BDNF) expression and cAMP response element-binding (CREB) phosphorylation in the hippocampus [37]. Although this study did not focus on the anti-inflammatory effects of soyasaponins, it still indicated that soyasaponins might be absorbed and transported to the brain and exerted its bioactivities. In 2016, Lin et al. showed that intraperitoneal administration of soyasaponin Ab (12.5, 25 and 50 mg/kg) inhibited LPS-induced acute lung injury in mice through attenuating lung pathological changes, edema, the expression of COX-2 and iNOS in lung tissues, as well as TNFа, IL-6, IL-1β and NO production in mice [38]. Furthermore, soyasaponin Ab activated liver X receptor alpha (LXRа) which is a member of the nuclear hormone receptor superfamily of ligand-activated transcription factors with anti-inflammatory effects. This study suggests that soyasaponins administrated by intraperitoneal injection can probably be distributed to lung and liver and play anti-inflammatory functions therein. Collectively, these studies indicate that soyasaponins do have in vivo anti-inflammatory bioactivities and our present study’s results further support this.

Secondly, the most important finding of this study is that soyasaponins (A_1_, A_2_ or I) can downregulate the LPS-increased expression of molecules in TLR4/MyD88 signaling pathway both in vivo and in vitro. LPS is one of the best studied immunostimulatory components of gram-negative bacteria. It has been widely used to establish inflammatory cell and animal models because it can induce both systemic and local tissue (e.g. liver, lung) inflammation [20, 28]. LPS stimulation can initiate TLR4 signaling by forming a complex with several proteins including TLR4, CD14 and MD-2. Following this, the intracellular MyD88-dependent signals are activated and transduced involving downstream molecules of TIRAP, IRAK-4, IRAK-1 and TRAF6 [20]. In the present study, soyasaponins (A_1_, A_2_ or I) significantly reduced the LPS-increased expression of MD-2, TLR4, MyD88, TIRAP and TRAF6, and phosphorylation of IRAK-4 and IRAK-1 in liver tissues of mice, which suggest the regulation of soyasaponins on TLR4/MyD88 signaling in vivo. To date, only one study from Lee et al. (2011) reported the effects of soyasaponins on TLR4/MyD88 signaling in vivo*.* They found that oral administration of soyasaponin Ab (10 and 20 mg/kg) to TNBS-induced colitic mice inhibited the expression of TLR4 and the phosphorylation of IRAK-1 in colon epithelial cells [13]. This is in accordance with part of our present results. In addition, a plant steroid saponin (Dioscin) with similar chemical structure to soyasaponin, has recently shown to alleviate LPS-induced inflammatory liver injury by decreasing the expressions levels of TLR4, MyD88, IRAK-1and TRAF6 in liver tissues of both mice and rat [28]. In contrast to the lack of investigations in vivo, quite a number of in vitro studies have investigated the molecular mechanisms (including TLR4 signaling) underlying soyasaponin’s anti-inflammatory bioactivities. It has been shown that soyasaponins can reduce inflammation by inhibiting the PI3K/Akt [7, 17], NF-κB [7, 11, 12, 17] and MAPKs [13, 18, 19] signaling pathway. NF-κB and MAPKs signaling which control the expression of pro-inflammatory cytokines are the downstream targets of TLR4 signaling pathway [20]. In LPS-stimulated mice peritoneal macrophages, soyasaponin Ab not only repressed the TLR4 expression and the IRAK-1 phosphorylation but also inhibited the binding of LPS to TLR4 [13]. Fussbroich et al. (2015) investigated the immune modulatory effect of soyasaponin I on TLR2- and TLR4-induced inflammation by stimulating the human acute myeloid leukemia-derived cell line MUTZ-3 with four different types of stimulators (the gram-negative *Escherichia coli,* gram-positive *Staphylococcus aureus,* LPS or peptidoglycans PGN). They found that the anti-inflammatory capacity of soyasaponin I was based on influencing both monocytic TLR2 and TLR4. Furthermore, soyasaponin I inhibited more effectively whole bacteria compared to solely LPS or PGN suggesting the whole bacteria are more sterically inhibited than the appropriate PAMPs by the unspecific binding of soyasaponin I to the outer membrane of MUTZ-3 cells [27]. This study broadens the anti-inflammatory mechanism of soyasaponin by blocking the binding of stimulators/ligands to TLR4 proposed by Lee et al [13]. In the study of Fussbroich et al., soyasaponin I had no effect on the expression of TLR2 and TLR4 in LPS- or PGN-stimulated MUTZ-3 cells [27]. The reason for explaining the lower effect of soyasaponin I on the expression of TLR levels in MUTZ-3 cells is probably that soyasaponin I had only weak regulation on MAPKs signaling which is the predominant regulator of TLR2 and TLR4 expression on these cells [12, 13, 27]. We previously showed that pre-treatment of soyasaponin Bb (40 μmol/L) did not alter the protein levels of TLR4, MyD88 and TRIF in LPS (for 15 min)-stimulated RAW264.7 macrophages [18]. It is well known that in in vitro inflammatory cell models, the treating concentration and duration of stimulators that used are key factors for regulating inflammation and its underlying molecular signaling. In macrophages, high-dose of LPS (> 10 ng/mL) is capable of opening up a flood gate of intracellular pathway through TLR4 and its co-receptors and eventually leading to the activation of NF-κB and MAPKs/AP-1 that contributing to the robust induction of pro-inflammatory mediators [39]. In the present study, we used 1 μg/mL of LPS to simulate RAW264.7 macrophages to establish an in vitro inflammatory cell model to further understand the regulation of soyasaponins (A_1_, A_2_ or I) on TLR4/MyD88-mediated inflammation. LPS stimulation for 10 min to 24 h did not produce significant change of the protein levels of MD-2, TLR4 and TIRAP. However, the effects of LPS stimulation on the expression of MyD88 and TRAF6, and the phosphorylation of IRAK-4 and IRAK-1 were dependent on the treatment duration. More specifically, LPS stimulation on macrophages for 30 min did not change the protein levels of MD-2, TLR4, TIRAP, MyD88, p-IRAK4 and p-IRAK1, which was not affected by pre-treatment of soyasaponins (A_1_, A_2_ or I). However, LPS stimulation for 30 min significantly increased the level of TRAF6, which could be blunted by soyasaponin I. Furthermore, LPS stimulation (for 1 h)-increased level of MyD88 was blocked by soyasaponins (A_2_ or I). LPS stimulation (for 3 h)-increased phosphorylation of IRAK4 and IRAK1 was blocked by all soyasaponins (A_1_, A_2_ or I). These results showed that soyasaponins can downregulate the expression of molecules in TLR4/MyD88 signaling pathway in inflammatory macrophages. Furthermore, soyasaponins (A_1_, A_2_, or I), similar to ST2825 (the MyD88 inhibitor), significantly reduced the MyD88 plasmid transfection-induced increase of MyD88 expression levels in HEK293T cells which normally express low MyD88. Together, these results suggest that MyD88 is probably the main target by soyasaponins (A_1_, A_2_, or I) in macrophages.

The final finding of this study is that soyasaponins (A_1_, A_2_, or I) can inhibit the recruitments of TLR4 and MyD88 into lipid rafts of liver tissue lysates of LPS-challenged inflamed mice. Lipid rafts is essential for the activation of TLR4/MyD88 signaling [30]. We previously found that soyasaponin Bb could reduce inflammation by inhibiting the recruitments of TLR4 and MyD88 into lipid rafts in murine macrophages in vitro [18]. Other plant-derived saponins with similar chemical structures to soyasaponins (glycyrrhizin and saikosaponin A) were also shown to be capable of suppressing the recruitment of TLR4 into lipid rafts [40–42]. These studies indicate that recruitments of molecules in TLR4 signaling pathway into lipid rafts may be the anti-inflammatory target by saponin compounds. To the best of our knowledge, the present study, for the first time, provides the in vivo evidence to support soyasaponins’ abilities to inhibit the recruitments of TLR4 and MyD88 into lipid rafts.

## Conclusion

**Fig. 6:**
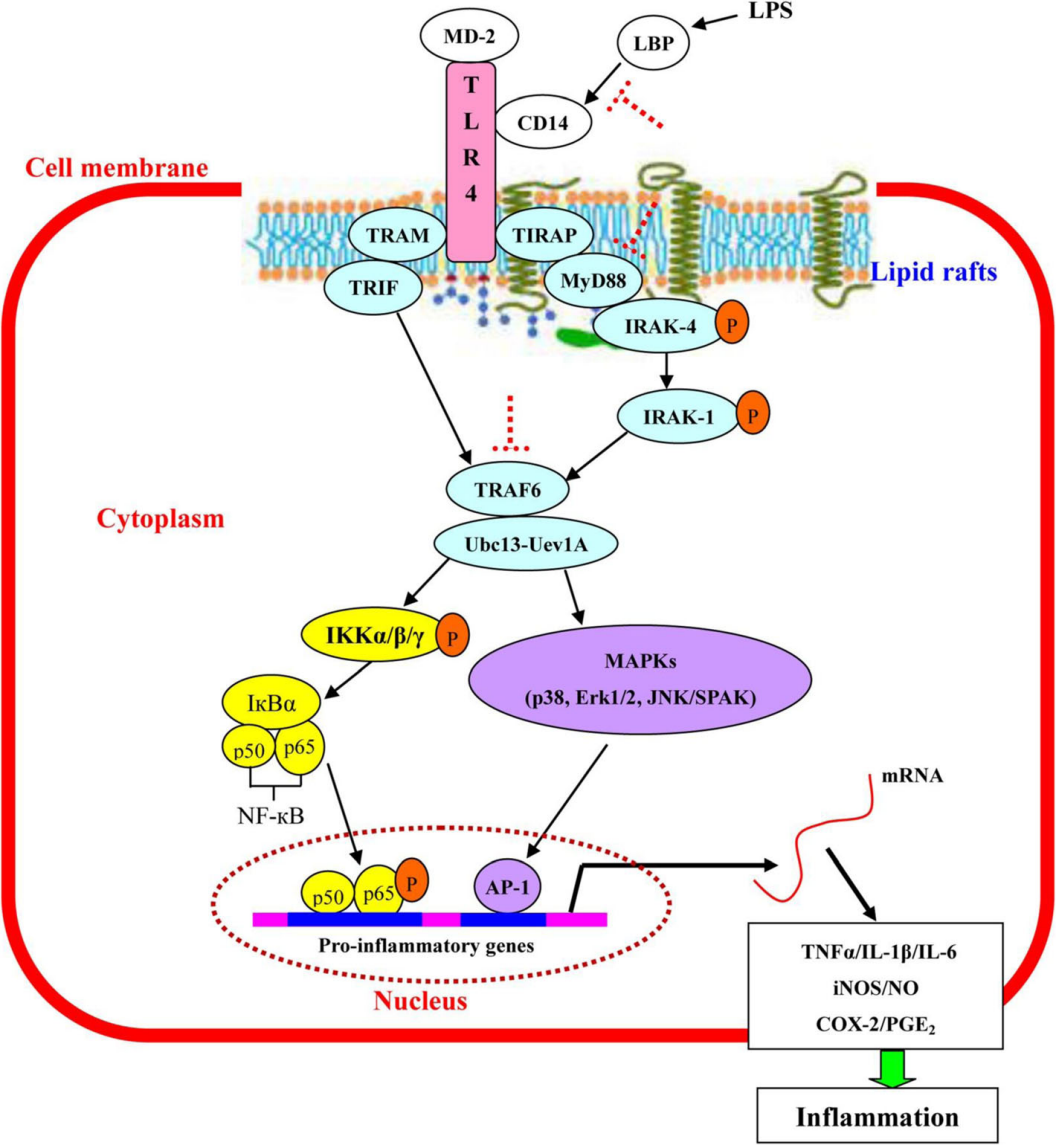
The schematic diagram of the mechanism of soyasaponins’ regulation on TLR4 signaling pathway. The red dotted T-shapes indicate the possible targets by which soyasaponins regulate TLR4 signals

This study shows that soyasaponins (A_1_, A_2_ or I) can reduce inflammation by downregulating MyD88 expression and suppressing the recruitments of TLR4 and MyD88 into lipid rafts. The present study provides novel understanding about the anti-inflammatory mechanism of soyasaponins. Based on the present study and previous studies from our and other labs, soyasaponins may regulate TLR4 signaling via three kinds of mechanisms: 1) to inhibit the binding of TLR4 to its ligands (e.g. LPS) [13, 27], 2) to suppress the recruitment of molecules (TLR4, MyD88 and TRIF) into lipid rafts [18], and 3) to reduce the expression of signal molecules [13] (the schematic diagram is shown in Fig. 6).

## Supplementary information

**Additional file 1 Table S1.** The inflammatory markers in serum of LPS-challenged ICR mice. **Table S2.** Effects of soyasaponins on the mRNA expression of inflammatory markers in the liver tissues of LPS-challenged ICR mice.

**Additional file 2 Figure S1.** Animal growth and feed intake of LPS-challenged inflammatory mice. Body weight and feed intake were monitored once a week. Body weight and feed intake were calculated during the establishment of LPS-induced inflammatory model in mice (A-B) and the soyasaponin intervention trial (C-D). Results reported are Means ± SD of *n* = 15 (C) and *n* = 3 (D) for each group. Data were statistically analyzed by using t-test or one-way ANOVA of SPSS software. **Figure S2.** The protein levels of molecules in TLR4/MyD88 signaling pathway in murine macrophages stimulated by LPS for different time. The murine RAW264.7 macrophages were treated with 1 μg/mL of LPS for different time (30 min to 24 h). The levels of molecules (MD-2, TLR4, MyD88, TIRAP, p-IRAK4, p-IRAK1 and TRAF6) in TLR4/MyD88 signaling pathway were determined by western blotting. Results reported are Means ± SD of three independent experiments. All data were statistically analyzed by using one-way ANOVA of SPSS software. *: *p* < 0.05 *v.s.* control.

## Data Availability

Materials used and data collected in this study are available from the corresponding author on reasonable request.

## Abbreviations

Akt: Protein kinase B
BDNF: Brain-derived neurotrophic factor
BW: Body weight
CD14: Cluster of differentiation 14
CD68: Cluster of differentiation 68
COX-2: Cyclooxygenase 2
CREB: CAMP response element-binding
DDMP: 2, 3-dihydro-2, 5-dihydroxy-6-methyl-4H-pyran-4-one
GAPDH: Glyceraldehyde-3-phosphate dehydrogenase
HFD: High fat diet
HPLC: High-performance liquid chromatography coupled with electrospray ionization tandem mass spectrometry
HRP: Horseradish peroxidase
ICAM-1: Intercellular cell adhesion molecule-1
ICR mice: Institute of cancer research mice
IKK: Inhibitor of nuclear factor kappa-B kinase
IL-1β: Interleukin 1 beta
IL-6: Interleukin 6
iNOS: Inducible nitric oxide synthase
IRAK1: Interleukin-1 receptor-associated kinase 1
IRAK4: Interleukin-1 receptor-associated kinase 4
IκB: Inhibitor of nuclear factor kappa-B
IκBα: Nuclear factor of kappa light polypeptide gene enhancer in B-cells inhibitor, alpha
LBP: LPS-binding protein
LPS: Lipopolysaccharide
LXRа: Liver X receptor alpha
MAPKs: Mitogen-activated protein kinases
MCP-1: Monocyte chemoattractant protein 1.
MD-2: Myeloid differentiation protein-2.
MES: 2-(N-morpholino)-ethanesulfonic acid.
MyD88: Myeloid differentiation factor 88.
NCDs: Non-communicable chronic diseases.
NF-κB: Nuclear factor kappa B.
NO: Nitric oxide.
PAMPs: Pathogen-associated molecular patterns.
PGE_2_: Prostaglandin E_2_.
PI3K: Phosphoinositide 3-kinase.
PMSF: Phenylmethylsulfonyl fluoride.
PVDF: Polyvinylidene fluoride.
RIPA: Radio-Immunoprecipitation Assay.
TAK1: Transforming growth factor-β-activated kinase 1.
TIRAP: Toll-interleukin1 receptor domain containing adaptor protein.
TLC: Thin-layer chromatography.
TLR4: Toll-like receptor 4.
TNBS: 3, 4, 5-trinitrobenzenosulfonic acid.
TNFα: Tumor necrosis factor alpha.
TRAF6: TNF receptor associated factor 6.
TRIF: Toll/interleukin 1 receptor domain-containing adaptor inducing IFN-β.
Ubc13: Ubiquitin-conjugating enzyme 13.
Uev1A: Ubiquitin-conjugating enzyme E_2_ variant 1 isoform A.
WHO: World Health Organization.
