# Supplementary material for: Soyasaponins reduce inflammation by downregulating MyD88 expression and suppressing the recruitments of TLR4 and MyD88 into lipid rafts
Source: BMC Complement Med Ther. 2020 Jun 3;20:167. doi: 10.1186/s12906-020-2864-2 (PMC7268359; doi:10.1186/s12906-020-2864-2)
Supplement: Supplementary file 1 — Additional file 1 Table S1. The inflammatory markers in serum of LPS-challenged ICR mice. Table S2. Effects of soyasaponins on the mRNA expression of inflammatory markers in the liver tissues of LPS-challenged ICR mice. [file 12906_2020_2864_MOESM1_ESM.docx]

**Table S1 The inflammatory markers in serum of LPS-challenged ICR mice**

| **Inflammatory markers** | **Control** | **LPS** |
| --- | --- | --- |
| TNFа (pg/mL) | 16.02±1.66 | 43.53±3.43 * |
| IL-6 (pg/mL) | 34.85±1.61 | 47.84±2.59 * |
| IL-1β (pg/mL) | - | - |
| NO (µmol/L) | 0.35±0.03 | 0.94±0.06 * |
| PGE_2_ (pg/mL) | 0.76±0.15 | 4.76±0.91 * |

The serum levels of TNFα, IL-6, IL-1β, PGE_2_ and NO were determined by commercial ELISA kits in ICR mice with intravenous injection of LPS via tail vein for 8 weeks. Results reported are Means ± SD of three pooled samples (n=3). For control group, one pooled sample was from the mixture of serum samples of three mice. For LPS group, one pooled sample was from the mixture of serum samples of ten mice. Data were statistically analyzed by using t-test of SPSS software. *: *p*<0.05 *v.s.* control. -: undetected.

**Table S2 Effects of soyasaponins on the mRNA expression of inflammatory markers in the liver tissues of LPS-challenged ICR mice**

|  | Control | LPS | LPS+Aspirin | LPS+10 mg/kgBW SS-A_1_ | LPS+20 mg/kgBW SS-A_1_ | LPS+10 mg/kgBW SS-A_2_ | LPS+20 mg/kgBW SS-A_2_ | LPS+10 mg/kgBW SS-I | LPS+20 μmol/kgBW  SS-I |
| --- | --- | --- | --- | --- | --- | --- | --- | --- | --- |
| TNFα | 1.00±0.16 | 8.56±1.37 * | 4.29±1.05 ^#^ | 5.39±1.54 ^#^ | 2.01± 0.61 ^#^ | 2.87±0.83 ^#^ | 1.57±0.42 ^#^ | 3.16±1.52 ^#^ | 1.29±0.14 ^#^ |
| IL-6 | 1.09±0.50 | 6.08±1.15 * | 3.01±1.00 ^#^ | 2.57±1.07 ^#^ | 1.52±0.16 ^#^ | 1.14±0.42 ^#^ | 1.60±0.27 ^#^ | 1.63±0.33 ^#^ | 2.61±0.51 ^#^ |
| IL-1β | 0.95±0.05 | 4.06±0.17 * | 2.01±0.22 ^#^ | 3.64±0.33 | 0.91±0.12 ^#^ | 1.23±0.02 ^#^ | 1.09±0.14 ^#^ | 1.38±0.15 ^#^ | 1.09±0.17 ^#^ |
| iNOS | 1.00±0.11 | 3.77±0.62 * | 1.62±0.24 ^#^ | 1.24±0.16 ^#^ | 2.06±0.23 ^#^ | 1.19±0.08 ^#^ | 1.30±0.35 ^#^ | 1.46±0.03 ^#^ | 0.92±0.27 ^#^ |
| COX-2 | 1.00±0.07 | 4.27±0.23 * | 1.67±0.38 ^#^ | 1.13±0.33 ^#^ | 1.06±0.08 ^#^ | 1.09±0.14 ^#^ | 1.20±0.06 ^#^ | 1.54±0.52 ^#^ | 2.15±0.19 ^#^ |

The relative mRNA expression (fold of control group) levels of TNFα, IL-6, IL-1β, iNOS, and COX-2 in liver tissues of LPS-challenged mice after intervention by aspirin or soyasaponins (A_1_, A_2_ or I) for 8 weeks were determined by real-time quantitative PCR. Data were statistically analyzed by using One-way ANOVA of SPSS software. Results presented are Means ± SD of 9 mice per group. *: *p*<0.05 vs. control, #: *p*<0.05 vs. LPS alone.
