# Supplementary figures and images for: Soyasaponins reduce inflammation by downregulating MyD88 expression and suppressing the recruitments of TLR4 and MyD88 into lipid rafts
Source: BMC Complement Med Ther. 2020 Jun 3;20:167. doi: 10.1186/s12906-020-2864-2 (PMC7268359; doi:10.1186/s12906-020-2864-2)

## Slide 1
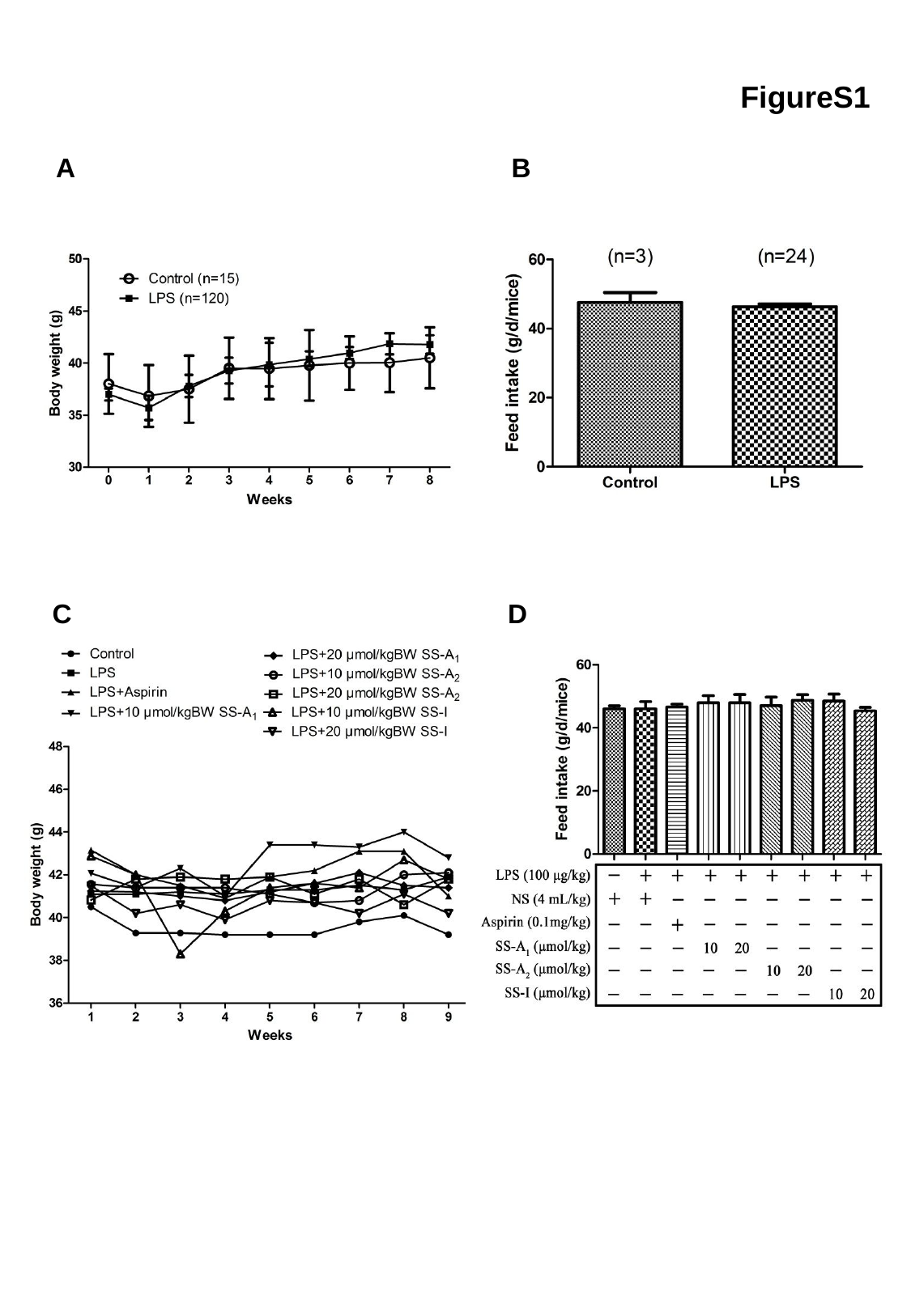

FigureS1
A
B
C
D

## Slide 2
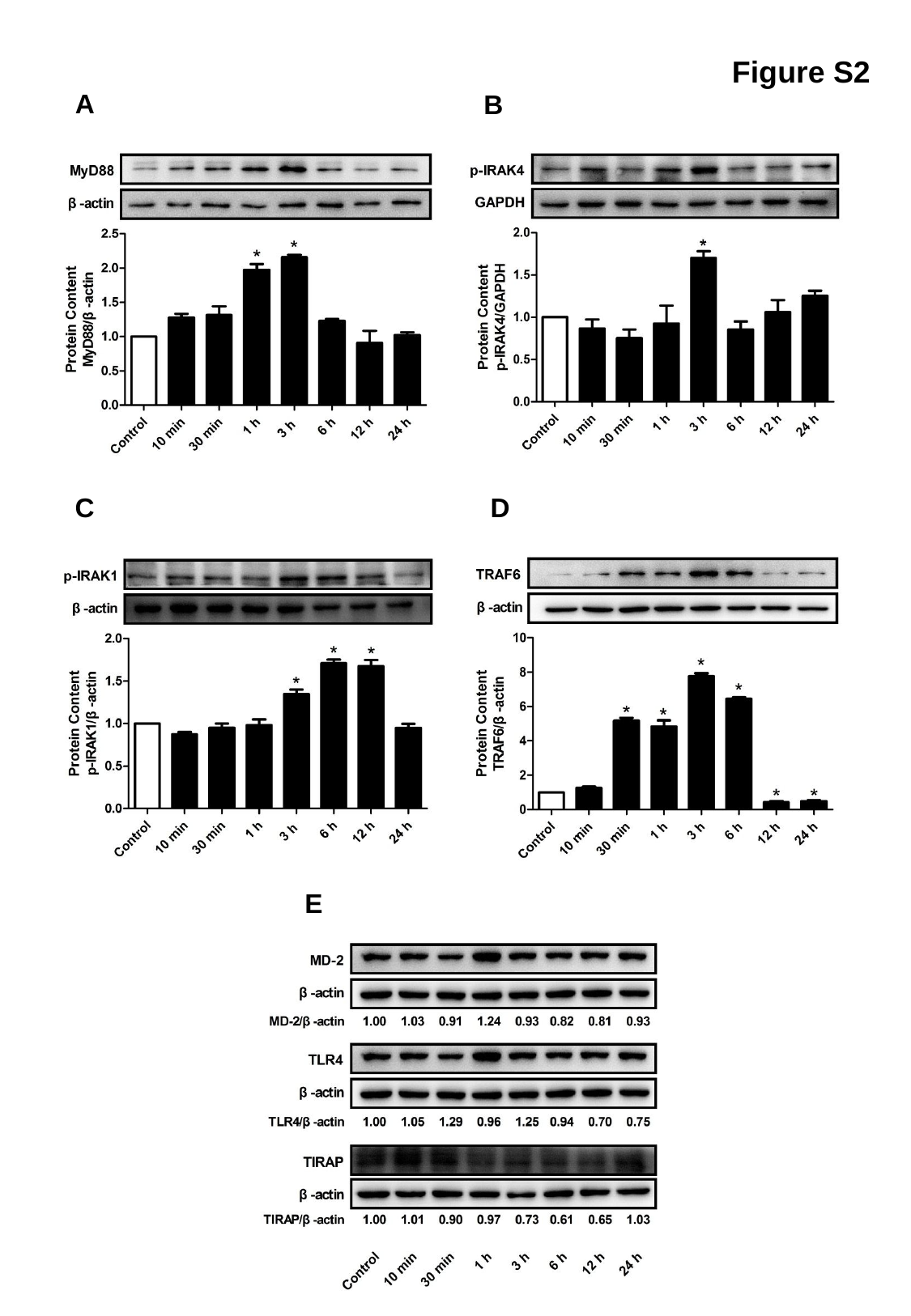

Figure S2
A
B
C
D
E

Supplement: Supplementary file 2 — Additional file 2 Figure S1. Animal growth and feed intake of LPS-challenged inflammatory mice. Body weight and feed intake were monitored once a week. Body weight and feed intake were calculated during the establishment of LPS-induced inflammatory model in mice (A-B) and the soyasaponin intervention trial (C-D). Results reported are Means ± SD of n = 15 (C) and n = 3 (D) for each group. Data were statistically analyzed by using t-test or one-way ANOVA of SPSS software. Figure S2. The protein levels of molecules in TLR4/MyD88 signaling pathway in murine macrophages stimulated by LPS for different time. The murine RAW264.7 macrophages were treated with 1 μg/mL of LPS for different time (30 min to 24 h). The levels of molecules (MD-2, TLR4, MyD88, TIRAP, p-IRAK4, p-IRAK1 and TRAF6) in TLR4/MyD88 signaling pathway were determined by western blotting. Results reported are Means ± SD of three independent experiments. All data were statistically analyzed by using one-way ANOVA of SPSS software. *: p < 0.05 v.s. control. [file 12906_2020_2864_MOESM2_ESM.ppt]
